# Late onset asymptomatic pancreatic neuroendocrine tumor – A case report on the phenotypic expansion for MEN1

**DOI:** 10.1186/s13053-017-0070-0

**Published:** 2017-07-21

**Authors:** Charu Kaiwar, Sarah K. Macklin, Jennifer M. Gass, Jessica Jackson, Eric W. Klee, Stephanie L. Hines, John A. Stauffer, Paldeep S. Atwal

**Affiliations:** 1 Center for Individualized Medicine, Mayo Clinic, Scottsdale, AZ 85259 USA; 2 Department of Clinical Genomics, Mayo Clinic, Jacksonville, FL 32224 USA; 3 Department of Neuroscience, Mayo Clinic, Jacksonville, FL 32224 USA; 4 Center for Individualized Medicine, Mayo Clinic, Jacksonville, FL 32224 USA; 5 Department of Health Sciences Research, Mayo Clinic, Division of Biomedical Statistic and Informatics, Rochester, MN 55905 USA; 6 Department of Clinical Genomics, Mayo Clinic, Rochester, MN 55905 USA; 7 Center for Individualized Medicine, Mayo Clinic, Rochester, MN 55905 USA; 8 Department of Internal Medicine, Mayo Clinic, Jacksonville, FL 32224 USA; 9 Department of General Surgery, Mayo Clinic, Jacksonville, FL 32224 USA

## Abstract

**Background:**

Multiple endocrine neoplasia type 1 (MEN1) is a hereditary cancer syndrome associated with several endocrine as well as non-endocrine tumors and is caused by mutations in the *MEN1* gene. Primary hyperparathyroidism affects the majority of MEN1 individuals by age 50 years. Additionally, *MEN1* mutations trigger familial isolated hyperparathyroidism. We describe a seemingly unaffected 76-year-old female who presented to our Genetics Clinic with a family history of primary hyperparathyroidism and the identification of a pathogenic *MEN1* variant.

**Case Presentation:**

The patient was a 76 year-old woman who appeared to be unaffected. She had a family history of a known *MEN1* pathogenic variant. Molecular testing for the known *MEN1* mutation c.1A > G, as well as, biochemical testing, MRI of the brain and abdomen were all performed using standard methods. Molecular testing revealed our patient possessed the *MEN1* pathogenic variant previously identified in her two offspring. Physical exam revealed red facial papules with onset in her seventies, involving her cheeks, nose and upper lip. Formerly, she was diagnosed with rosacea by a dermatologist and noted no improvement with treatment. Clinically, these lesions appeared to be facial angiofibromas. Brain MRI was normal. However, an MRI of her abdomen revealed a 1.5 cm lesion at the tail of the pancreas with normal adrenal glands. Glucagon was mildly elevated and pancreatic polypeptide was nearly seven times the upper limit of the normal range. The patient underwent spleen sparing distal pancreatectomy and subsequent pathology was consistent with a well-differentiated pancreatic neuroendocrine tumor (pNET).

**Conclusions:**

Age-related penetrance and variable expressivity are well documented in families with MEN1. It is thought that nearly all individuals with MEN1 manifest disease by age 40. We present a case of late-onset MEN1 in the absence of the most common feature, primary hyperparathyroidism, but with the presence of a pNET and cutaneous findings. This family expands the phenotype associated with the c.1A > G pathogenic variant and highlights the importance of providing comprehensive assessment of *MEN1* mutation carriers in families that at first blush may appear to have isolated hyperparathyroidism.

## Background

Multiple endocrine neoplasia 1 (MEN1) or Wermer’s Syndrome is an autosomal dominant disorder caused by germ line mutations in the *MEN1* gene located in the region 11q13.1 [1]. *MEN1* is a tumor suppressor gene that encodes for the protein, Menin. This 610 amino acid protein is localized within the nucleus, has two functional nuclear localization signals and represses JunD mediated transcription. It is expressed in a variety of tissues and is conserved across species from Drosophila to *Homo sapiens*. Menin is proposed to act as a tumor suppressor, as *MEN1-*associated tumors follow Knudson’s “two-hit hypothesis” [2, 3]. MEN1 is characterized by the presence of a combination of tumors of the parathyroid glands, anterior pituitary, and Islet cells of the pancreas. In addition, tumors of the adrenal cortex, facial angiofibromas, carcinoid tumors, collagenous tumors, and lipomatous tumors have also been described. A clinical diagnosis of MEN1 is made in cases where there is occurrence of two or more primary MEN1 tumor types, or where there is one of the MEN1-associated tumors and a family member with a clinical diagnosis of MEN1. About 10–20% patients with familial MEN1 do not have a detectable pathogenic variant in the *MEN1* gene [4]. The MEN1 phenotype is highly variable even within families and no genotype-phenotype correlations have been made to date [5]. The clinical presentation and age of diagnosis vary by the type of tumor. Primary hyperparathyroidism is the most common first presentation in patients with MEN1, followed by Pancreatic Neuroendocrine Tumors (pNET) and Pituitary tumors (PIT) [6]. In fact, primary hyperparathyroidism is diagnosed in a majority of patients before the diagnosis of MEN1. The age of diagnosis varies from the first to the fifth decade, median age being 37 years for patients with MEN1 pathogenic variants and 55 years for patients without MEN1 pathogenic variants [6].

In this report we describe a case of MEN1 where diagnosis of hyperparathyroidism in family members prompted the 76 years old asymptomatic proband to seek a genetics referral. The report expands on the phenotype of this syndrome and also highlights the phenotypic variability associated with it.

## Case Presentation

**Fig. 1:**
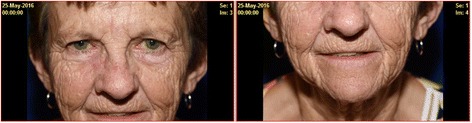
76 years old female proband with facial angiofibromas

**Fig. 2:**
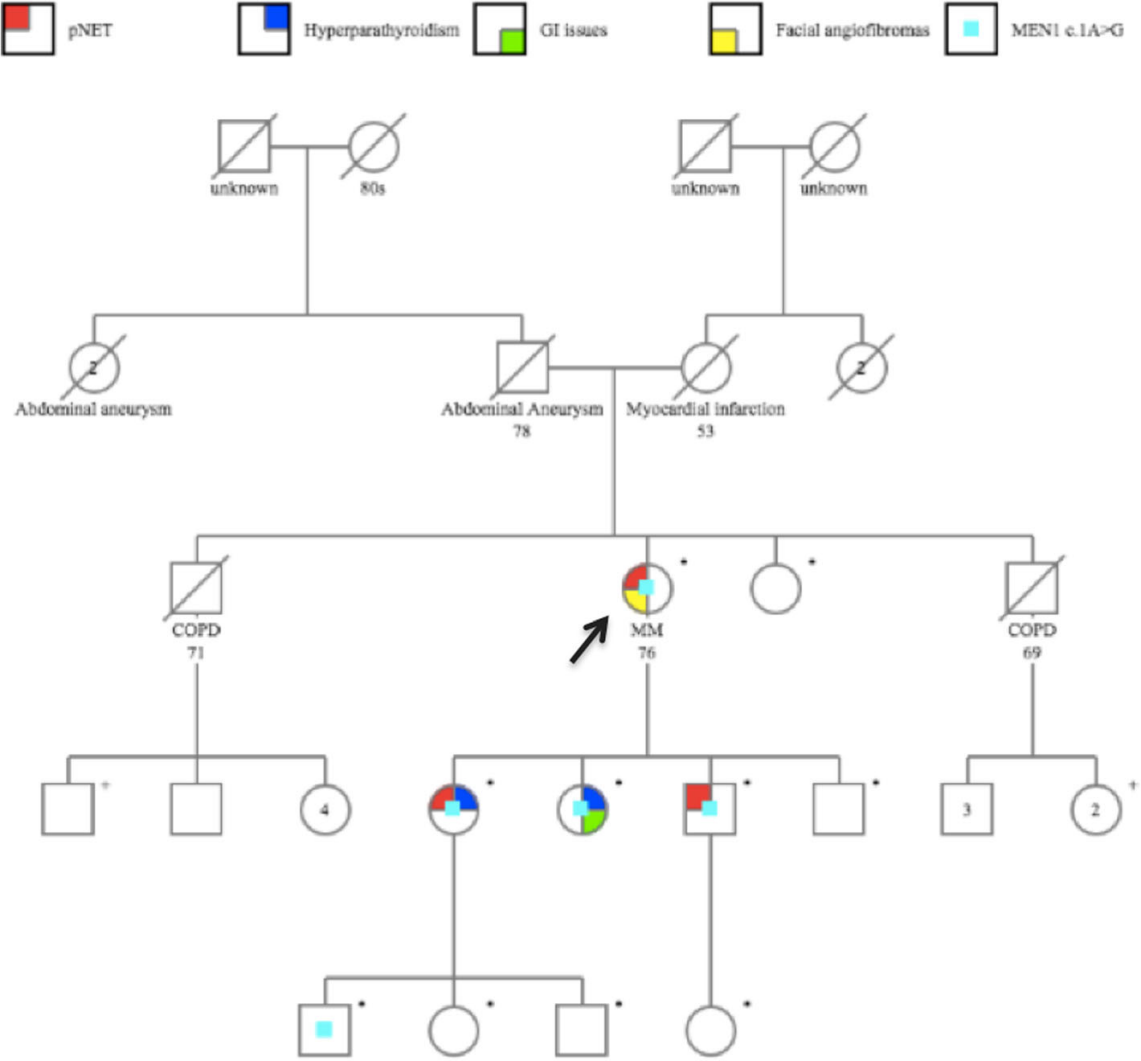
Pedigree- the black arrow indicates the proband. The symbol * denoted individuals who underwent genetic testing for MEN1. The symbol + denotes individuals who intend to get tested in the upcoming year

A 76 year- old female was referred to our genetics clinic due to a family history of MEN1 in two children and a familial pathogenic variant in *MEN1*. She did not have a diagnosis of any of the “three P” tumors classic to MEN1 - parathyroid, pituitary or pancreas. Her past medical history was significant for hypothyroidism and osteoporosis, for which she was being appropriately treated with levothyroxine and intermittent bisphosphate replacement. She also had a history of lymphoma, which was treated with Cyclophosphamide, Hydroxy doxorubicin, Vincristine (Oncovin), and Prednisone (CHOP) regimen. Surgical history was notable for a cholecystectomy. A complete physical examination revealed no other abnormalities, except for the presence of red papules on the cheeks, nose, vermillion border of the upper lip and chin. The patient reported that these papules had been present for the past 4–5 years. On closer examination by the geneticist, these papules were diagnosed as facial angiofibromas (Fig. 1). Her family history was significant for two daughters with MEN1 diagnosed at 48 and 53 years of age and a son with hypercalcemia and elevated parathyroid hormone levels (Fig. 2). Following standard practice guidelines for first-degree relatives of patients with MEN1, she was offered targeted genetic testing for the known familial pathogenic variant in *MEN1*. Screening for endocrine tumors was also performed with MRIs of the head and abdomen (for pituitary and pancreatic tumors). Plasma Ca^++^, parathyroid hormone levels (PTH), prolactin and Insulin like Growth Factor (IGF-1) levels, as well as fasting gastrointestinal tract hormone profile, (that includes measurement of gastrin, glucagon, vasointestinal polypeptide, pancreatic polypeptide, chromogranin A, and insulin with fasting glucose levels) were also performed.

**Fig. 3:**
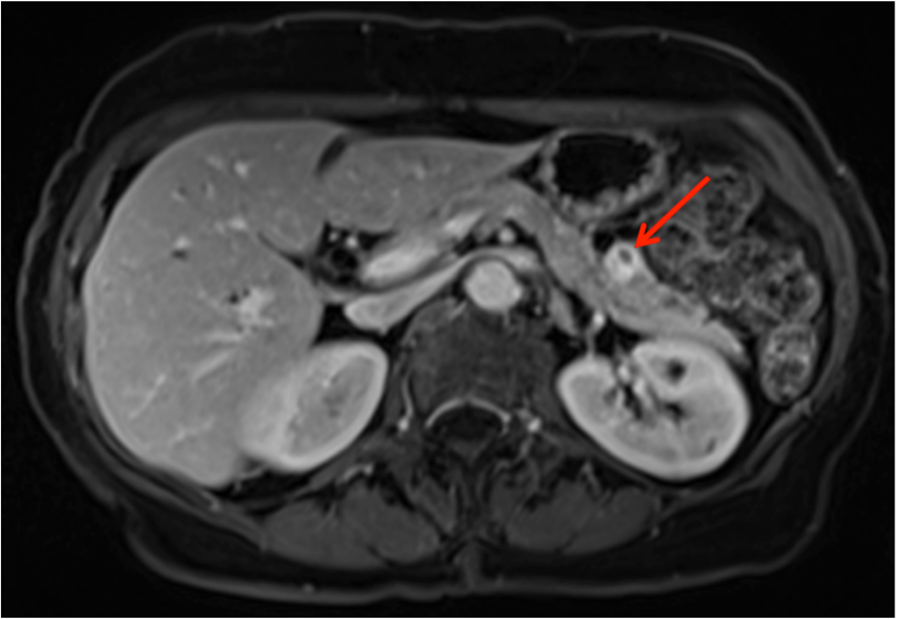
Axial venous phase MRI showing an enhancing 1.5 cm cystic mass consistent with pancreatic neuroendocrine tumor in the tail of the pancreas (*red arrow*)

Genetic testing was positive for the familial pathogenic variant *MEN1* c.1A > G, that results in the loss of the translation initiation codon. The imaging revealed an solitary enhancing, partially cystic pancreatic tail lesion suggestive of a neuroendocrine tumor (Fig. 3) measuring 1.1 × 1.3 × 1.5 cm. Endoscopic ultrasound was then performed which confirmed the lesion in the tail of the pancreas and no other lesions were identified after thorough inspection of the entire pancreas. Laboratory tests revealed elevated pancreatic polypeptide of 2210 pg/mL, while Chromogranin A was normal at 52 ng/mL. The gastrointestinal hormone profile was significant only for slightly elevated glucagon at 89 pg/mL. Given the isolated pancreatic lesion, the patient was considered a candidate for surgery. She underwent an uncomplicated laparoscopic spleen-preserving distal pancreatectomy and discharged the next day. Pathology confirmed it to be a 1.4 × 1.0 cm well-differentiated neuroendocrine tumor, positive for keratin, synaptophysin and chromogranin stains. Regional lymph nodes were negative for metastatic disease and the Ki67 index was less than 2%. Incidentally, adenomatosis and an additional pNET measuring 2 mm in size were identified.

## Discussion and conclusions

The *MEN1* c.1A > G pathogenic variant was first reported in 2002 [7] in a family with a history of isolated hyperparathyroid hormone levels and hypercalcemia, diagnosed with familial isolated hyperparathyroidism (FIHP). The pathogenic variant segregated with the disease in 3 out of 8 members tested across two generations, while one tested individual had no symptoms and was deemed at risk. Additional literature reports [8] of this pathogenic variant have been published with the affected patients having variable symptoms like parathyroid and pancreatic islet neoplasia with or without other tumors.

Other reports involving this start codon of *MEN1,* such as c.2 T > A [9], also exhibit the intrafamilial phenotypic variability associated with variation in this amino acid residue, as different family members with the same pathogenic variant are affected by different endocrine tumors. In our case, the proband was asymptomatic on presentation, despite having two daughters that became symptomatic at the earlier ages of 44 and 48 years. One was diagnosed with hyperparathyroidism and pNET while the other had a diagnosis of hypercalcemia and gastrointestinal symptoms. Both tested positive for the same *MEN1* pathogenic variant, which prompted our asymptomatic 76 year-old patient to seek testing. Most patients with MEN1 have shown biochemical manifestations by age 40–50 years [10–12]. Specifically pancreatic neuroendocrine tumors (pNET) are associated with an earlier age of onset in patients with MEN1 mutations than those without MEN1 mutations [10, 12–14]. In our case, the patient did not exhibit any biochemical symptoms of MEN1, in spite of being followed by endocrinology for a history of hypothyroidism and osteoporosis. The pancreatic tumor was found at 76 years of age only after an MRI of the abdomen was performed as standard protocol for screening MEN1 family members. This tumor could be correlated as being non-functional given the increased chromogranin A staining.

Regarding the patient’s co-existence of MEN1 and lymphoma, there has been a prior case report [15] demonstrating this, and indeed it is an interesting observation. Whilst no known association or mechanism exists in the medical literature, to our knowledge this is only the second case of MEN1 and lymphoma in the same patient. This may be purely co-incidental however given the lack of other cases.

Once our proband tested positive for the pathogenic variant, she was encouraged to share her results with other family members. Her son also tested positive for the pathogenic variant and was found to have a pancreatic tumor on subsequent screening at age 57 years. This highlights the value of medical genetics consultation in patients with MEN1 so that family members at risk can be regularly screened for the endocrine tumors.

This case report brings to attention the variable age of diagnosis for MEN1, the phenotypic heterogeneity of MEN1, even within the same family, and a proband that was asymptomatic until age 76 years, barring facial angiofibromas. The report signifies the importance of medical genetics consultation for diagnosis of MEN1, and, careful screening for MEN1 associated tumors when a pathogenic variant is identified.

## Abbreviations

CHOP: Cyclophosphamide, Hydroxy doxorubicin, Vincristine (Oncovin), and Prednisone
FIHP: familial isolated hyperparathyroidism
IGF-1: Insulin like Growth Factor
MEN1: Multiple Endocrine Neoplasia
PIT: Pituitary Tumor
pNET: Pancreatic Endocrine Tumor
PTH: Parathyroid hormone
